# Optogenetic stimulation reveals distinct modulatory properties of thalamostriatal vs corticostriatal glutamatergic inputs to fast-spiking interneurons

**DOI:** 10.1038/srep16742

**Published:** 2015-11-17

**Authors:** Giuseppe Sciamanna, Giulia Ponterio, Georgia Mandolesi, Paola Bonsi, Antonio Pisani

**Affiliations:** 1 University of Rome “Tor Vergata”, Dept. of Systems Medicine, via Montpellier 1 -00133, Rome; 2 Fondazione Santa Lucia IRCCS, Neurophysiology and Plasticity lab, via Fosso di Fiorano 64 -00143, Rome

## Abstract

Parvalbumin-containing fast-spiking interneurons (FSIs) exert a powerful feed-forward GABAergic inhibition on striatal medium spiny neurons (MSNs), playing a critical role in timing striatal output. However, how glutamatergic inputs modulate their firing activity is still unexplored. Here, by means of a combined optogenetic and electrophysiological approach, we provide evidence for a differential modulation of cortico- vs thalamo-striatal synaptic inputs to FSIs in transgenic mice carrying light-gated ion channels channelrhodopsin-2 (ChR2) in glutamatergic fibers. Corticostriatal synapses show a postsynaptic facilitation, whereas thalamostriatal synapses present a postsynaptic depression. Moreover, thalamostriatal synapses exhibit more prominent AMPA-mediated currents than corticostriatal synapses, and an increased release probability. Furthermore, during current-evoked firing activity, simultaneous corticostriatal stimulation increases bursting activity. Conversely, thalamostriatal fiber activation shifts the canonical burst-pause activity to a more prolonged, regular firing pattern. However, this change in firing pattern was accompanied by a significant rise in the frequency of membrane potential oscillations. Notably, the responses to thalamic stimulation were fully abolished by blocking metabotropic glutamate 1 (mGlu1) receptor subtype, whereas both acetylcholine and dopamine receptor antagonists were ineffective. Our findings demonstrate that cortical and thalamic glutamatergic input differently modulate FSIs firing activity through specific intrinsic and synaptic properties, exerting a powerful influence on striatal outputs.

The striatum is the primary input nucleus of the basal ganglia (BG) and receives massive glutamatergic innervation from both the cerebral cortex^1^,^2^,^3^,^4^ and by a number of thalamic nuclei^4^,^5^. Corticostriatal and thalamostriatal inputs to medium spiny neurons (MSNs) and cholinergic interneurons (ChIs) have been recently characterized by both immunohistochemical and electrophysiological approaches, demonstrating peculiar, distinct synaptic properties^4^,^6^,^7^,^8^. However, cortex and thalamus provide also a dense glutamatergic innervation to other subtypes of striatal interneurons^4^,^9^. In particular, parvalbumin-containing fast-spiking interneurons (FSIs) receive inputs from cerebral cortex and from intralaminar thalamic nuclei^10^,^11^. Despite such evidence, the synaptic properties of these glutamatergic inputs to FSIs have not been characterized yet.

FSIs represent less than 2% of the entire striatal neuronal population but their widely divergent output and the mutual electrotonic coupling make these interneurons the main source of inhibitory GABAergic control onto MSNs^11^,^12^ and a crucial factor in modulating the whole striatal output. FSIs show also peculiar firing properties^13^,^14^: indeed, with current injection they exhibit an intermittent firing pattern consisting of periods of high frequency spike trains abruptly interrupted by periods of quiescence and subthreshold oscillation^15^,^16^. The mechanisms underlying such peculiar pattern is not well understood, although previous data demonstrated that membrane oscillations can trigger the intermittent spike bursts^15^, and that intermittent pattern requires the presence of the low-threshold slowly inactivating Kv1 current^16^. In addition, this firing pattern observed *in vitro* is believed to reflect the gamma oscillation observed in the striatum *in vivo*^15^,^16^.

In the present study, by means of a novel optogenetic approach we performed a detailed electrophysiological characterization of the corticostriatal and thalamostriatal inputs onto FSIs. We demonstrate that excitatory synapses originating from cortex and thalamus differently modulate FSIs excitability.

## Results

### Characterization of corticostriatal and thalamostriatal synapses onto FSIs

**Figure 1:**
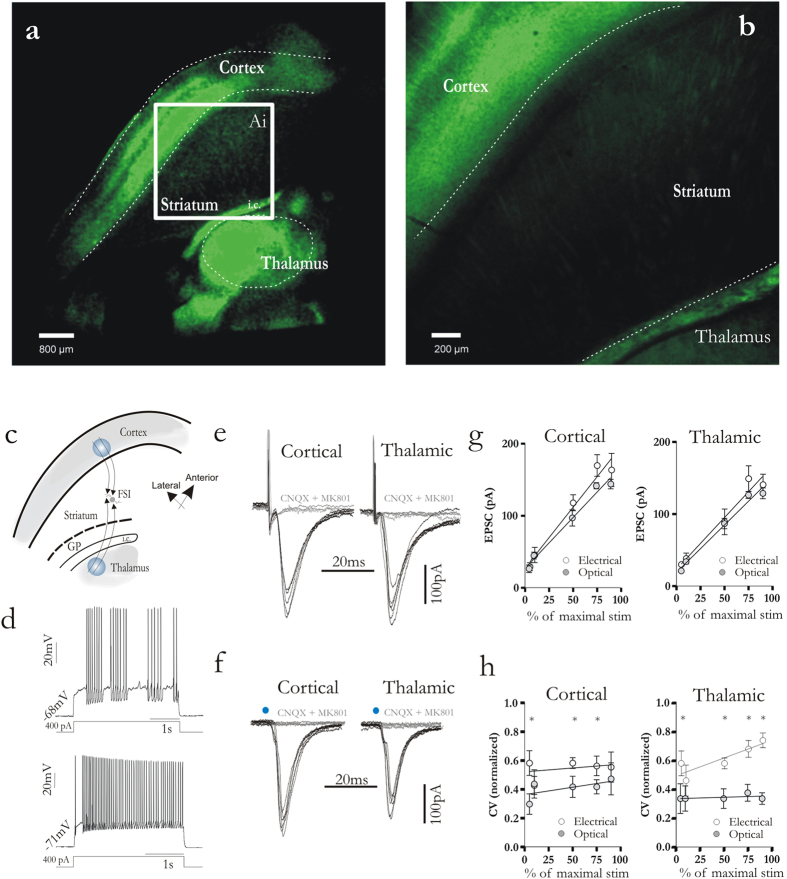
Optogenetic characterization of corticostriatal and thalamostriatal synapses of striatal FSIs. (**a**) Representative confocal images from parahorizontal brain slices of mice expressing the ChR2 -EYFP fusion protein. The low magnification shows ChR2-eYFP expression (green) mainly in the cortical neurons of layer V and neurons in the thalamus (scale bar: 800 μm). (**b**) High magnification showing fluorescent glutamatergic fibers also in the striatum (scale bar: 200 μm). (**c**) Composite drawn image of parahorizontal slice used for electrophysiological recording. Blue spots highlight the site of light stimulation for corticostriatal and thalamostrial stimulation. Shaded areas represent ChR2-carrying region. (**d**) Representative traces of peculiar firing pattern of FSIs induced by depolarizing current injection (400 pA, 3.5 s) (**e,f**), EPSCs (black traces) elicited by cortical and thalamic stimulation by means of electrical (**e**) and optical (**f**) pulses (blue dots indicate when light pulse was delivered). Bath application of CNQX (10 μM) and MK801 (30 μM) fully abolished EPSCs evoked both by cortical and thalamic stimulation (grey traces) (**g,h**), Summary graph of input-output relationship and coefficient of variation (CV) calculated for both thalamostriatal and corticostriatal synapses. Error bars indicate SEM. Asterisks indicate statistical significance.

The physiological properties of corticostriatal and thalamostriatal synapses onto striatal FSIs were studied by means of both optical and electrical stimulation (Fig. 1a–c). Fifty-six neurons from 39 animals were identified as FSIs. Candidate neurons were chosen according to their morphology (DIC images) and to their electrophysiological membrane properties (Fig. 1d). Under current-clamp conditions intracellular current injections (250 pA, 1 s duration) induced a maximal firing frequency (>100 Hz). Long-duration (5 s) positive current injection induced bursts of action potentials spaced out by quiescent intervals of variable duration that were randomly interrupted by other bursts of action potentials (Fig. 1d). All these properties are in line with previous reports^12^,^13^,^14^,^15^,^17^. Electrical stimulation (10–25 volt, 20 μs, 10 s intervals) from both cortex and thalamus induced, in whole-cell voltage-clamped FSIs, a clear excitatory post-synaptic current (EPSC) (Fig. 1e; corticostriatal EPSCs: 108 ± 11 pA n = 17; thalamostriatal EPSCs: 89 ± 20 pA n = 14, 50% of maximal stimulation, see methods). Likewise, light pulses (470 nm; 3 ms duration, 10 s intervals) were able to evoke corticostriatal and thalamostriatal EPSCs (Fig. 1f, corticostriatal EPSCs: 96 ± 11 pA; thalamostriatal EPSCs: 86 ± 7 pA; n = 15, 50% of maximal stimulation, see methods). All experiments were performed in the presence of GABA_a_ receptor antagonist (picrotoxin, 10 μM). Slice preincubation with 10 μM CNQX and 30 μM MK-801 fully abolished the evoked responses, demonstrating that only glutamatergic fibers were stimulated (Fig. 1e,f, grey traces). Input-output relationship (increasing stimulation intensity was calculated as % of the stimulation able to induce an action potential, see methods) showed comparable results between electrical and optical stimulation at both cortical and thalamic synapses (Fig. 1g). However, the related coefficient of variation (CV) was significantly different. EPSCs elicited by electrical stimulation at both corticostriatal and thalamostriatal fibers had a higher CV value compared to stimulation triggered by light pulse (Fig. 1h). These data suggest that light pulse provides a more reliable stimulation, avoiding non-specific current spread.

### Corticostriatal and thalamostriatal synapses differ in paired pulse ratio (PPR) and NMDA/AMPA ratio

**Figure 2:**
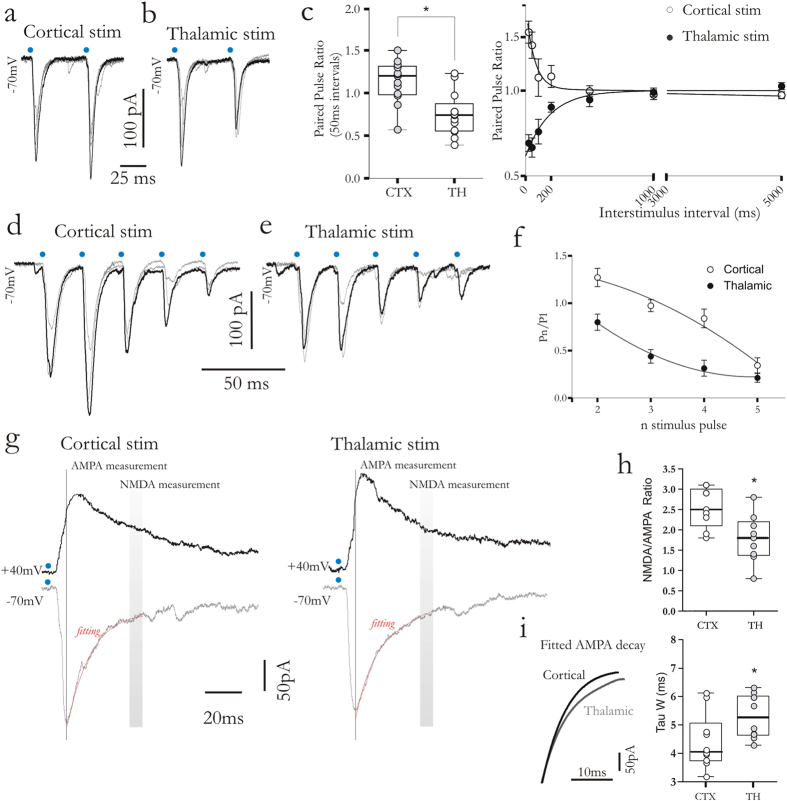
Corticostriatal and thalamostriatal synapses exhibit distinct short-term synaptic plasticity and NMDA/AMPA current ratio. (**a,b**) Corticostriatal and thalamostriatal EPSCs elicited by paired-pulse paradigm (50 ms interstimulus interval, ISI). Blue dots highlight when optical stimulation was delivered. (**c**) Summary box plot (left) of paired pulse ratios (PPRs) showing facilitation at corticostriatal synapses and depression at thalamostriatal synapses. The graph (right) of PPRs is plotted against increasing interstimulus intervals for corticostriatal (white circles) and thalamostriatal (black circles) stimulation. **(d,e)** EPSCs induced by trains of optical stimulation at cortical and thalamostriatal afferents (5 light pulse, 20 Hz). (**f**) Graph summarizing data reported in (**d**,**e**). EPSCs amplitude was normalized to the first EPSC amplitude and plotted against stimulus number for corticostriatal and thalamostriatal stimulation (respectively white and black circles). (**g**) Representative, superimposed EPSCs traces following corticostriatal and thalamostriatal optical stimulation, recorded while neurons were held at a holding potential (HP) of −70 mV and +40 mV, respectively. Black line and grey bars indicate windows used to measure peak of AMPA-mediated currents (1 ms) and peak of NMDA-mediated currents (10 ms), both at +40 mV. (**h**) Box-plot of NMDA/AMPA current ratios elicited by cortical and thalamostriatal stimulation. (**i**) Normalized fitted decay curve of AMPA-mediated current. Note a slower decay time at thalamic synapses. The plot shows *tau-weighted* time constant (*T*w). Error bars indicate SEM. Asterisks indicate statistical significance.

Short-term synaptic plasticity is a transient change in the efficacy of synaptic transmission. When a synapse is stimulated in rapid succession, the second post-synaptic response can be larger or smaller than the first one (termed paired-pulse facilitation, PPF or depression PPD, respectively). Paired-pulse ratio (PPR) represents a reliable tool to study short-term plasticity. FSIs were recorded in whole-cell voltage-clamp mode, whereas cortical or thalamic afferents were optically activated. Short conditioning intervals (50 ms, Fig. 2a) induced a PPF at corticostriatal synapses. With longer duration intervals (ms), PPR returned to 1 (Fig. 2a,c) (p < 0.05, Mann–Whitney test). Conversely, at thalamostriatal synapses, a PPD was elicited at short intervals (50 ms, Fig. 2b). PPR returned close to 1 with longer duration intervals (Fig. 2b,c) (p < 0.05, Mann–Whitney test). To investigate more in detail the short-term dynamics at both synapses, cortical and thalamic afferents were stimulated with train of pulses consisting of 5 light flashes at 20 Hz. In line with previous data^6^, corticostriatal synapses presented a short-term facilitation (at first and second pulse) followed by a constantly depressed response (Fig. 2d,f). Conversely, following the first pulse, thalamostriatal synapses exhibited a steady depression (Fig. 2e,f). The postsynaptic abundance of AMPA and NMDA receptors is another important property of glutamatergic fibers. Thus, we measured the NMDA/AMPA current ratios at both synapses (Fig. 2g). We estimated the kinetic of the AMPA component by stimulating while the cell was held at a holding potential of –70 mV. The slice was then stimulated while the cell was held at +40 mV. Such depolarized holding potential reveals the NMDA component of the EPSC. The NMDA/AMPA ratio was smaller at thalamostriatal synapses than at corticostriatal synapses (Fig. 2g,h) (corticostriatal, 2.55 ± 0.16, n = 13; thalamostriatal 1.78 ± 0.19, n = 14; p = 0.01, Mann-Whitney test), demonstrating a more abundant AMPA-mediated current at thalamostriatal synapses. Moreover, fitted curve of AMPA-mediated current (Fig. 2i) demonstrated that EPSCs evoked by thalamostriatal synapses decayed more slowly than those elicited by corticostriatal stimulation with a higher weighted time constant (τw) (Fig. 2i, box plot) (corticostriatal, 4.4 ± 0.3, n = 13; thalamostriatal 5.31 ± 0.24, n = 14; p = 0.03; Mann–Whitney test).

### Corticostriatal and thalamostriatal synapses exhibit distinct release properties

**Figure 3:**
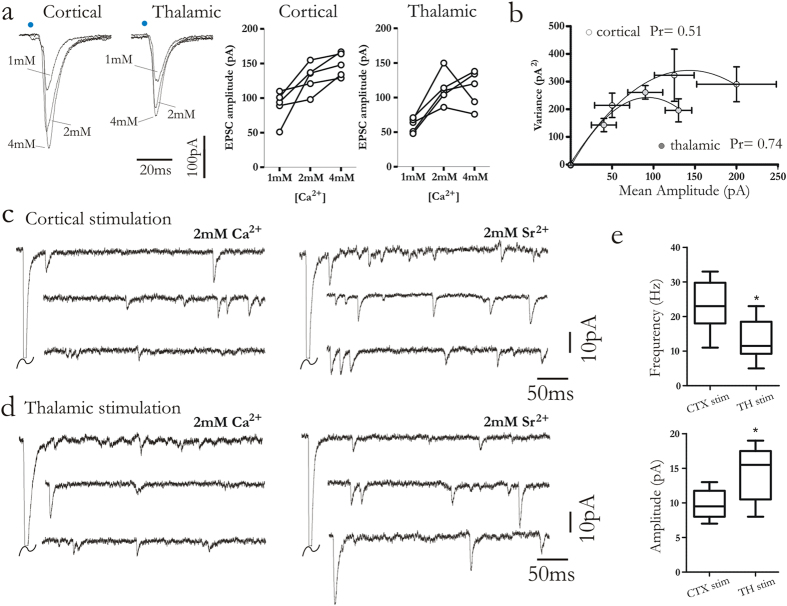
Distinct release probability at corticostriatal and thalamostriatal synapses. (**a,b**) Superimposed EPSCs showing cortical and thalamic stimulation responses in the presence of 1 mM, 2 mM and 4 mM Ca^2+^ in the external solution. Plots (right) summarize the increased EPSC amplitude depending on external Ca^2+^ concentrations. (**b**) Average variance-mean curve for thalamic and corticostriatal synaptic responses. Data were fitted with a parabolic function to estimate the release probability (Pr). At 2 mM Ca^2+^concentration estimated Pr for thalamostriatal synapses (grey circles) was 0.74. For corticostriatal synapses (white circle) estimated Pr was 0.51. Error bars indicate SEM. Asterisks indicate statistical significance. (**c,d**) Representative traces recorded in 2 mM Ca^2+^(left) and in 2 mM Sr^2+^/0 mM Ca^2+^(right) after cortical and thalamic stimulation in order to induce asynchronous release. Experiments were performed in the presence of 30 μM MK-801. (**e**) Box-plots summarizing asynchronous release frequency and amplitude after optical stimulation of cortical and thalamic synapses. Blue dots highlight when light pulse was delivered. Error bars indicate SEM. Asterisks indicate statistical significance.

To explore the differences in release probability, cortical and thalamostriatal synapses were studied, by modifying the concentration of extracellular Ca^2+^[Ca^2+^] (Fig. 3a). A low concentration (from 2 mM to 1 mM) caused a depression of EPSC amplitude both at thalamostriatal and corticostriatal synapses. By increasing the extracellular Ca^2+^(from 2 mM to 4 mM) a rise of EPSC amplitude was recorded both at thalamostriatal and corticostriatal synapses (Fig. 3a). To estimate the presynaptic release probability (P_r_) we performed variance-mean analysis^18^ (Fig. 3b). EPSC amplitude was recorded at three different extracellular [Ca^2+^] (external [Ca^2+^] = 1 mM, 2 mM and 4 mM). Briefly, (see methods), the variance of the EPSC amplitude was plotted against the mean amplitude at each concentration forming a parabola. The degree of parabolic curvature estimates P_r_, and the limiting slope under low release conditions estimates the Quantal release (Q_av_)^19^. P_r_ at thalamostriatal synapses was significantly higher than corticostriatal synapses (Fig. 3b) (Thalamostriatal P_r_ = 0.74, n = 9; corticostriatal P_r_ = 0.51 n = 9; p = 0.03, Wilcoxon signed rank). Conversely, no significant change was found in the estimated Q_av_ between corticostriatal and thalamostriatal synapses (Thalamostriatal Q_av_ = 6.9 n = 9; Corticostriatal Q_av_ = 7.1 n = 9; p > 0.05 Wilcoxon signed rank). A further approach to assess synaptic properties is to record miniature EPSCs (mEPSCs). In order to detect quantal events at corticostriatal and thalamostriatal FSIs synapses, extracellular recording solution was modified by replacing Ca^2+^with Sr^2+^and both pathways were optically stimulated^6^,^8^ With this approach, corticostriatal and thalamostriatal quantal events could be studied in the same FSI (Fig. 3c,d).

The modal frequency of asynchronous, quantal EPSCs (qEPSCs) measured after thalamic stimulation was significantly lower than after cortical stimulation (Fig. 3c–e) (thalamic stim. 13.30 ± 1.86 Hz; *n* = 13; cortical 23.40 ± 2.2 Hz; n = 13, 23 cells; *p* = 0.07, Mann–Whitney test). These results further indicate a relatively higher probability of transmitter release at thalamostriatal synapses than at corticostriatal synapses. Moreover, the amplitude of qEPSCs evoked by thalamic stimulation was significantly higher than the synaptic events measured after cortical stimulation (thalamic stim., 14.25 ± 1.38 pA; cortical stim., 9.75 ± 0.75 pA; *p* = 0.03, Mann–Whitney test) suggesting that thalamic synapses have higher vesicle size than corticostriatal synapses (Fig. 3e bottom).

### Distinct modulation of corticostriatal and thalamostriatal synapses during high-frequency firing activity of FSIs

**Figure 4:**
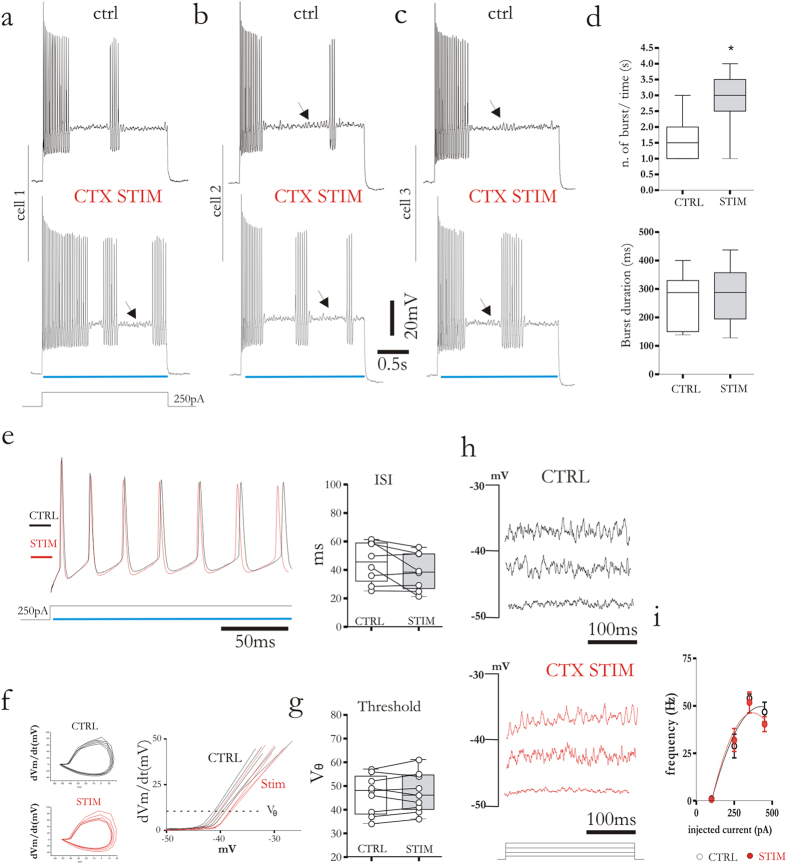
Corticostriatal stimulation modulates evoked firing activity of FSIs. (**a–c**) Representative traces of evoked firing activity induced by depolarizing current injection (250 pA, 2.5 s) from three distinct cells. Traces were recorded in control condition (upper) and during simultaneous corticostriatal stimulation by repetitive flashlights (lower). Note a higher frequency of bursts during optical stimulation. (**d**) Box plots summarizing the effects of corticostriatal stimulation on burst number and duration. (**e**) Superimposed traces (left) of evoked firing activity (250 pA current pulse) recorded before (black trace) and during (red trace) corticostriatal stimulation. The ISI plot confirms the lack of statistical significance of the change in firing rate between the two groups. (**f**) Phase plots of d*V*_m_/d*t* against membrane voltage calculated from evoked action potential in control condition (black) and during corticostriatal stimulation (red). Superimposed enlarged traces (right) show where threshold voltage (V_θ_) for action potential generation was defined (dotted line). (**g**) Box plot summarizes the effects of the corticostriatal stimulation on the threshold for action potential generation. (**h**) Representative membrane oscillations recorded from the same cell in control condition and during corticostriatal stimulation by means of repetitive light pulses. Oscillations were triggered by injection of depolarizing current steps (100–450 pA). (**i**) Plot summarizing the effect of corticostriatal stimulation on oscillation frequency. Blue lines indicate when light pulse was delivered. Error bars indicate SEM. Asterisks indicate statistical significance.

**Figure 5:**
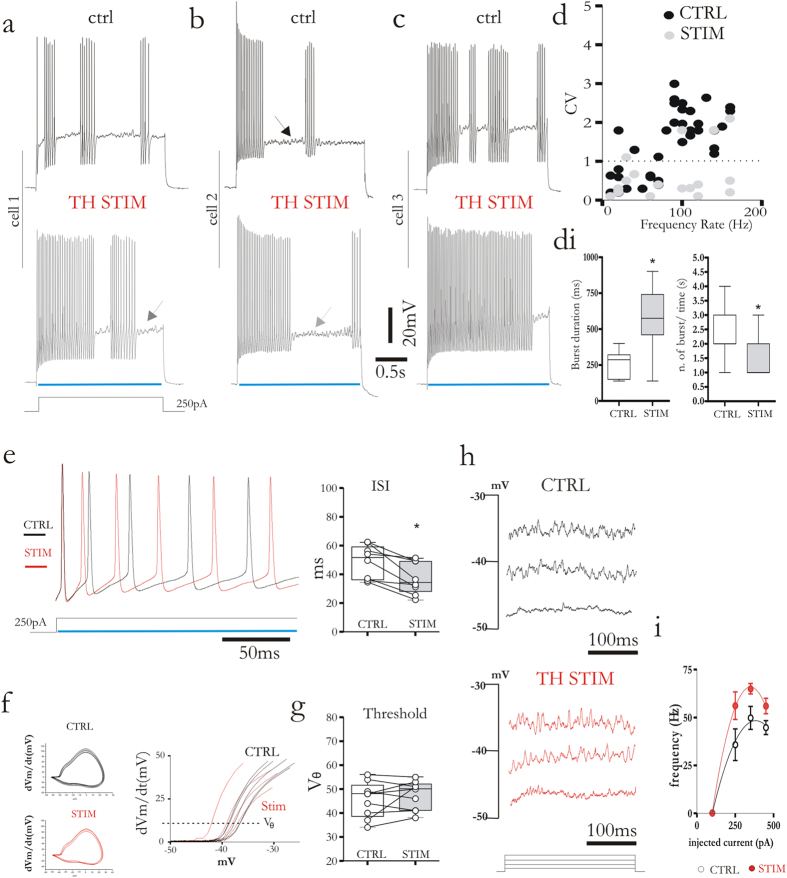
Thalamostriatal stimulation modulates evoked firing activity of FSIs. (**a–c**) Representative traces recorded from three distinct cells, showing their firing activity induced by depolarizing current injection (250 pA, 2.5 s). Traces were recorded in control condition (upper) and during simultaneous thalamostriatal stimulation (lower). Note a more prolonged and regular firing pattern during thalamic fiber stimulation. (**d**) Graph of mean CV against mean evoked firing rate in control (black circles) and during thalamostriatal stimulation (grey circles). Dotted line corresponds to Poisson firing (CV = 1, random spike train). Note that stimulation protocol shifts CV towards lower value (~0) indicative of a more regular firing pattern. Box-plot show increased burst duration after thalamic stimulation (**e**), Superimposed traces of evoked firing activity (250 pA current pulse, left) recorded before (black trace) and during (red trace) thalamostriatal stimulation. Box plot (right) shows a decreased inter-spike interval (ISI) following stimulation. (**f**) Phase plots of d*V*_m_/d*t* against membrane voltage calculated from evoked action potential in control condition (black) and during thalamostriatal stimulation (red). Superimposed enlarged traces (right) show where threshold voltage (V_θ_) for action potential generation was defined (dotted line). (**g**) Box plot shows that thalamostriatal stimulation does not affect V_θ_. (**h**) Representative membrane oscillations recorded from the same cell in control conditions and during thalamostriatal synapses stimulation by means of repetitive light pulses. Oscillations were triggered by injection of depolarizing current steps of increasing amplitude (100–450 pA). (**i**) Plot summarizes the effect of thalamostriatal stimulation on oscillations frequency. Blue lines highlight when light pulse was delivered. Error bars indicate SEM. Asterisks indicate statistical significance.

FSIs were silent at their resting membrane potential, but in response of near-threshold current pulses the cells exhibited their peculiar firing pattern including burst of action potentials spaced by quiescent periods (“stuttering” firing)^16^, accompanied by voltage-dependent sub-threshold membrane potential oscillations (Figs 4 and 5a–c; black arrows). The source of FSIs firing pattern is not completely understood, but previous reports showed that membrane subthreshold oscillations can trigger a new burst of action potentials^15^. Moreover, the occurrence of stuttering regime requires the presence of the low-threshold slowly inactivating Kv1 current^16^. However, thus far there is no evidence regarding a possible role played by synaptic inputs in modulating FSI firing pattern. To address this issue, FSIs were recorded in current-clamp configuration and a 2.5 s duration positive current pulse (250 pA) was injected to trigger action potential discharge (Fig. 4a–c, upper). A simultaneous train of light pulses (470 nm, 3 ms, 10 Hz) was delivered to activate corticostriatal synapses (Fig. 4a–c, lower). This stimulation caused a clear change in the firing pattern, inducing a net increase of the number of bursts, without changing their duration (Fig. 4d). Instantaneous firing frequency was not affected by corticostriatal stimulation with not significant changes in the inter-events intervals (Fig. 4e). Yet, cortical fibers stimulation did not affect the spike thresholds (V_θ_) determined by constructing phase plots dV_m_/dt against V_m_ (Fig. 4g). As expected, the quiescent period between two bursts was characterized by voltage membrane oscillations. Thus, by means of multiple depolarizing current steps (100, 250, 350, 450 pA) we tested the frequency of the oscillation and measured the effect of corticostriatal stimulation (Fig. 4h–i). Oscillations appeared if the neurons was depolarized by ~30 mV from resting membrane potential. The frequency of the oscillations recorded without corticostriatal stimulation (51.8 ± 1.56 Hz, after a 350 pA depolarizing current step, n = 8) was not significantly different compared to the frequency recorded during optical stimulation at all depolarizing steps (Fig. 4h,i; 50.6 ± 1.67 Hz, n = 9; p = 0.95, Mann-Whitney test).

We replicated the same set of experiments to study how thalamostriatal synapses could modulate current-evoked firing frequency in FSIs (Fig. 5). During optical stimulation of thalamic fibers (Fig. 5a–c, lower) FSIs showed an increased number of action potentials during a depolarizing current step. Indeed, thalamic stimulation switched the firing pattern towards a prolonged, more regular activity with a partial loss of the peculiar stuttering firing (Fig. 5a–c). Population graph (Fig. 5d) displaying mean CV vs. mean firing rate demonstrated that in control condition (black dots) evoked firing activity of FSIs have a CV > 2 typical of an irregular/bursting firing pattern^20^. Conversely, during thalamic stimulation CV values (Fig. 5d, grey dots) were close to 0.5, indicating a more regular firing rate. Moreover, stimulation of thalamic fibers significantly increased the frequency of evoked action potentials (Fig. 5e; ctrl 45 ± 19 Hz Th stim: 67 ± 15 Hz, p = 0.3, Mann-Whitney test) (250 pA; 2.5 sec). A significant lower inter-spike interval corroborates this result (Fig. 5e). Phase plots dV_m_/dt against V_m_ showed no significant changes in action potential threshold (V_θ_) during thalamic optical stimulation (Fig. 5f,g). Finally, we also tested whether membrane oscillations were affected by thalamic stimulation. Contrarily to the corticostriatal synapses, activation of thalamic fibers by means of light pulses significantly increased the frequency of voltage membrane oscillation triggered by a series of depolarizing current steps (100, 250, 350, 450 pA) (Fig. 5h,i) (350 pA injected current, ctrl: 50.8 ± 2.27 Hz, Th stim: 73 ± 0.59 Hz n = 8 p = 0.45, Mann-Whitney test). Membrane oscillation amplitude was affected neither by cortical or thalamic stimulation (CTX stim: from: 3.1 ± 1.27 pA to 3.88 ± 1.57 ; Th stim: from 3.5 ± 0.59 pA to 2.9 ± 1.06 n = 8 p > 0.45, Mann-Whitney test).

### Thalamic or cortical modulation of evoked firing activity of FSIs does not depend upon dopamine and acetylcholine receptors

**Figure 6:**
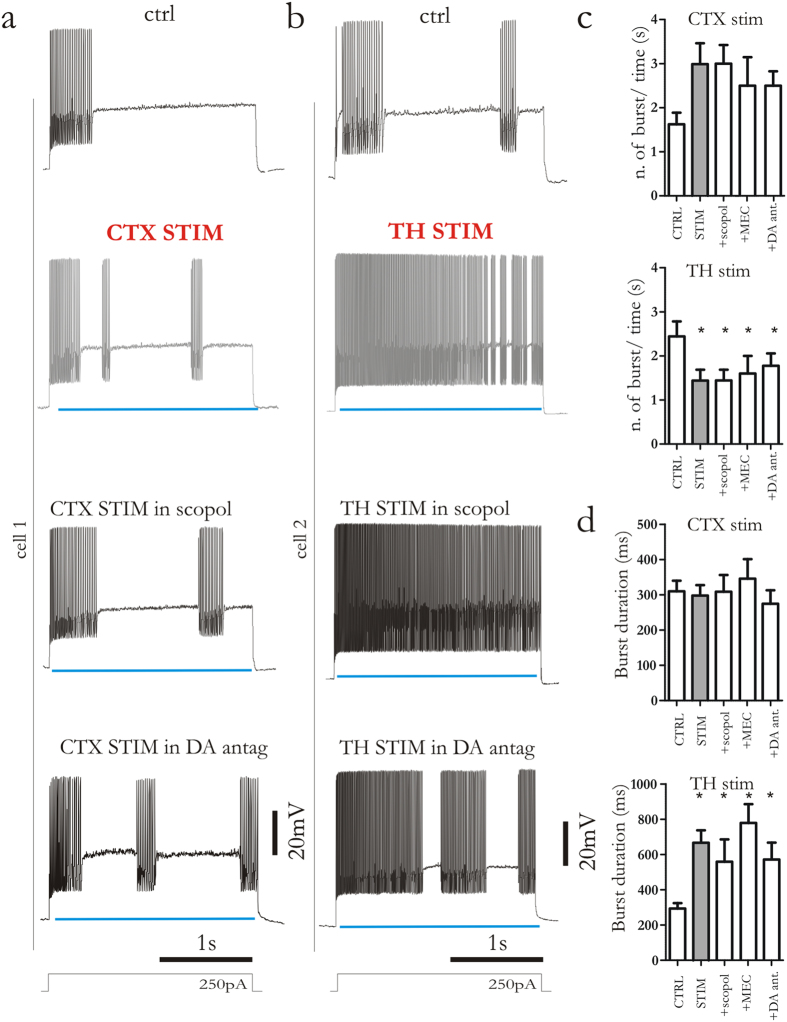
Firing pattern in FSIs induced by thalamic or cortical activation does not require dopamine and acetylcholine receptor activation. (**a,b**) Representative traces from two distinct cells showing evoked firing activity triggered by depolarizing current injection (250 pA, 2.5 s). Traces were recorded in control condition (upper line), during cortical and thalamostriatal stimulation (middle line, grey traces) and during bath application of acetylcholine (ACh) muscarinic receptor antagonist scopolamine (3 μM) or a combination of D1- and D2-like dopamine (DA) receptor antagonists (SCH23390, 10 μM; sulpiride, 3 μM) together with cortical and thalamostriatal stimulation (bottom lines). (**c,d**) Bar plots summarize the effect of the nicotinic receptor antagonist mecamylamine (MEC), scopolamine and DA antagonists. Blue lines highlight when light pulse was delivered. Error bars indicate SEM. Asterisks indicate statistical significance.

Glutamatergic fibers from thalamus and cortex also innervate striatal cholinergic interneurons (ChIs)^7^,^8^,^10^,^21^. Thus, activation of thalamic and cortical synapses would result in acetylcholine (ACh) release that might, in turn, influence firing activity of FSIs. Of interest, ACh directly excites FSIs through nicotinic receptor activation^22^. To investigate this hypothesis, we measured the effect of thalamic and cortical stimulation in the presence of either ACh muscarinic or nicotinic receptor antagonists (scopolamine, 3 μM, 10 min, and mecamylamine hydrochloride, MEC, 10 μM, 10 min, respectively) (Fig. 6a,b). When muscarinic and nicotinic blockers were applied, cortical stimulation still induced the increase in the number of bursts (Fig. 6a,c,d). Moreover, both scopolamine and MEC were unable to counteract the shift of evoked firing pattern triggered by thalamic stimulation (Fig. 6b–d). Glutamatergic stimulation of ChIs could also lead to an indirect dopamine (DA) release, through presynaptic nicotinic receptor activation^23^. D1-like receptor activation has been demonstrated to depolarize FSI^24^. Thus, to test whether DA release could determine changes in FSI spiking activity (Fig. 6a,b), slices were preincubated with a combination of D1- and D2-like DA receptor antagonist (SCH23390, 10 μM and sulpiride, 3 μM, 15 min, respectively). Blockade of DA receptors was not able to affect firing pattern changes triggered by both thalamic or cortical stimulation. Indeed, neither number of bursts nor burst duration were affected (Fig. 6a–d).

### Thalamic modulation of firing activity requires mGlu1 receptor activation

**Figure 7:**
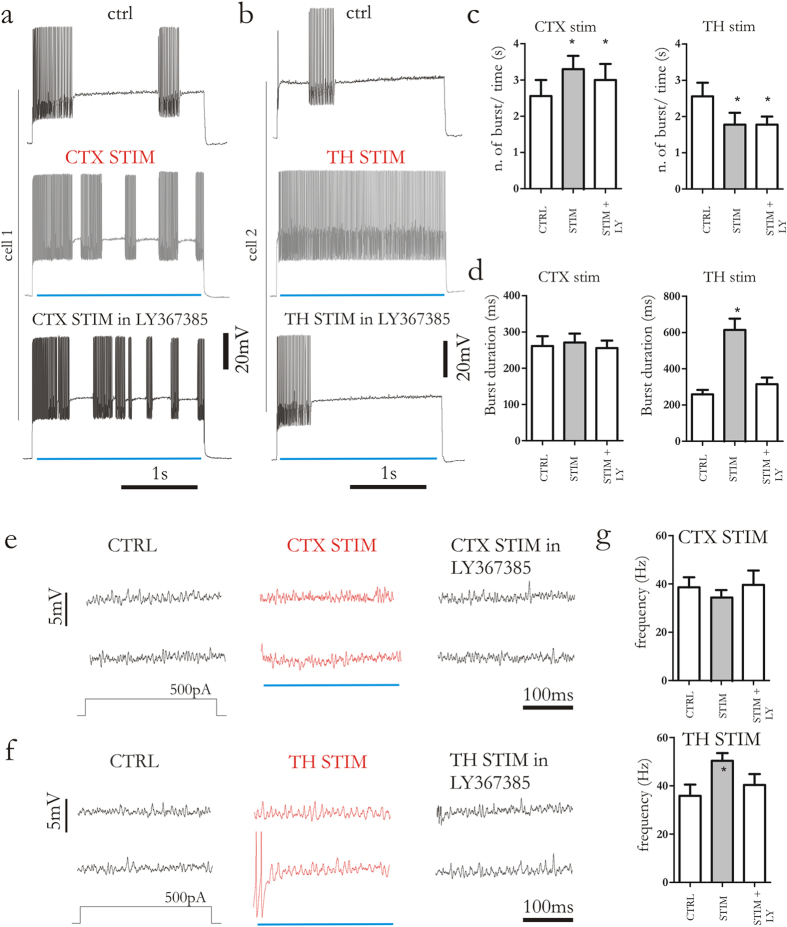
Thalamic modulation of firing activity requires mGlu1 receptors. (**a,b**) Representative traces from two distinct cells showing evoked firing activity triggered by depolarizing current injection (250 pA, 2.5 s). Traces were recorded in control condition (upper line), during cortical and thalamostriatal stimulation (middle line, grey traces) and during bath-application of LY367385 (30 μM, 10 min) together with cortical and thalamostriatal stimulation (bottom line). (**c,d**) Bar plots summarize the effect of LY367385. Note that LY367385 reverts the effect of thalamostriatal but not corticostriatal stimulation on evoked firing activity. (**e,f**), Representative membrane oscillations recorded in control conditions, during cortical and thalamostriatal synaptic stimulation (red traces) and during synaptic stimulations coupled with bath-application of LY367385 (30 μM, 10 min). Oscillations were triggered by injection of depolarizing current steps (500 pA, 2.5 s). (**g**) Bar plots summarize the effect of LY367385 on membrane oscillations during cortical and thalamostriatal stimulation. Blue lines indicate when light pulse was delivered. Error bars indicate SEM. Asterisks indicate statistical significance.

FSIs express both group I metabotropic glutamate (mGlu) receptor subtypes (mGlu1 and mGlu5 receptors), and activation of these receptors leads to a robust membrane depolarization. Of note, mGlu1, but not mGlu5 receptor activation underlies this excitatory effect^25^. Moreover, cortico- and thalamostriatal synapses show a distinct localization of group I mGlu receptors. In particular, presynaptic mGlu1 receptors have been found predominantly on thalamostriatal afferents^4^. Thus, we measured the effect of thalamic and cortical stimulation in the presence of the selective mGlu1 receptor antagonist, LY367385 (10–30 μM, 10 min). The increase in bursts number recorded upon photostimulation of corticostriatal synapses was not affected by bath-application of LY367385 (Fig. 7a). Contrarily, the changes in firing pattern triggered by thalamic photostimulation were fully prevented by blocking mGlu1 receptors (Fig. 7b). Additionally, LY367385 was also able to block the increase in frequency of subthreshold membrane oscillations upon thalamic photostimulation (Fig. 7f,g). Membrane oscillations recorded during cortical stimulation were not affected by LY367385 (Fig. 7e,g).

## Discussion

The main findings of the present study show that thalamostriatal afferents differently modulate FSIs excitability compared to glutamatergic cortical inputs. In detail: i) corticostriatal synapse activation induces a postsynaptic facilitation, whereas thalamostriatal fibers stimulation cause a postsynaptic depression; ii) Thalamostriatal synapses exhibit more prominent AMPA-mediated currents than corticostriatal synapses, and an increased release probability; iii) our optogenetic approach demonstrates that evoked burst-pause activity is markedly increased when cortical fibers are activated. Conversely, thalamic fiber activation triggers a higher frequency of subthreshold oscillations together with a shift towards prolonged but more regular firing pattern; iv) mGlu1 receptors are critically involved in the responses of FSIs to thalamic, but not cortical stimulation.

Striatal neural population receives dense glutamatergic projections both from neocortex and thalamus^4^,^26^,^27^ and both anatomical and behavioural studies suggest fundamentally different functions of these pathways. FSIs are largely innervated by cortex^10^,^28^ and also by centromedian (CM) and parafascicular (PF) intralaminar nuclei^4^,^9^,^29^. To date, surprisingly few investigations have been carried out to explore the electrophysiological properties of both these glutamatergic inputs^6^,^30^. Exploring how they can modulate striatal circuit is crucial in understanding the basal ganglia physiology, but to date, both the anatomical complexity and some technical limitations have slowed down this target. In particular, although electrical stimulation represents the most commonly utilized method for neural stimulation, it has some inherent limitations. Conversely, optical stimulation can achieve excitation of specific cell populations without indirect and unwanted synaptic overlapping and recordings of neural response are not contaminated by the excitation stimulus^21^,^31^,^32^. Thus, we first performed a detailed comparison of synaptic currents evoked by electrical versus optical stimulation. We found that when optically stimulated, cortical and thalamic fibers elicited a more stable synaptic response with a significant lower coefficient of variation, as compared to electrically-evoked EPSCs. Some technical limitations should be acknowledged, and require a note of caution in the overall interpretation of our data. One central issue is the potential antidromic activation triggered by thalamic stimulation of corticostriatal and corticothalamic pathways that collateralize in the striatum^30^,^33^. However, previous anatomical investigations suggest that in a parahorizontal slice preparation, cortical fibers projecting first in the striatum and then to other brain structures, are rostrally segregated to the thalamic stimulation site^34^,^35^ and axons are preserved. Thus, although both the mouse model and the slice preparation we utilized suggest that photostimulation protocol recruits effectively two distinct afferent pathways, our approach cannot fully exclude stimulation of unintended fibers.

Our data show that optical stimulation induced clear and stable EPSCs at both cortico- and thalamostriatal synapses with not significant differences in size or kinetics. Conversely, short-term plasticity experiments show a facilitation at corticostriatal synapses (PPRs > 1) and a depression at thalamostriatal synapses (PPRs < 1). Further results suggesting substantial differences between cortico and thalamostriatal synapses of FSIs came from experiments modulating extracellular Ca^2+^concentration. By means of mean-variance analysis of the synaptic responses^18^ we demonstrated that synapses from thalamus have a higher release probability than corticostriatal synapses. This evidence was corroborated by studying asynchronous quantal release (qEPSCs). Frequency of qEPSCs was significantly higher after cortical stimulation than after thalamic stimulation supporting the idea of a significant difference in probability of transmitter release^6^. Moreover higher amplitude of qEPSCs recorded at thalamostriatal synapses suggest differences in vesicle size and/or postsynaptic AMPA-mediated current. In addition to differing in short-term plasticity, corticostriatal and thalamostriatal synapses diverged in their relative abundance of AMPA and NMDA ionotropic receptors. Indeed, our data demonstrate that at thalamostriatal synapses, the amplitude of AMPA-mediated currents was significantly greater than NMDA-mediated currents measured at corticostriatal synapses. AMPA receptors are highly dynamic ionotropic receptors and they mediate fast excitatory transmission^25^. The relative higher abundance of AMPA receptors together with high release probability imply that thalamic-mediated excitation may produce fast but rapidly recovering excitation in FSI. Conversely, cortical discharge can induce a robust NMDA-mediated depolarization leading to long-lasting modifications of synaptic strength.

*In vitro,* FSIs are electrophysiologically characterized by high firing frequency discharge in response to somatic current steps. They also exhibit periods of repetitive firing interrupted by periods of quiescence (stuttering firing) and subthreshold oscillations^15^,^16^,^36^. Moreover, in awake behaving animals the discharge of striatal FSI neurons is characterized by irregular fluctuations of the instantaneous firing rate with numerous high-frequency bursts, single spikes, and periods of silence^37^,^38^,^39^. While ionic mechanism underlying fast firing activity were partly elucidated^16^, it is not currently known if the FSI firing variability is determined by synaptic inputs that these interneurons receive under physiological conditions. Interestingly, we found that cortical stimulation increases the number of bursts triggered by simultaneous depolarizing current step, with no change in firing rate. Conversely, activation of thalamic fibers reduced the peculiar stuttering activity by increasing the evoked firing frequency and shifting the firing towards a prolonged more regular firing pattern.

Intermittent spike bursts are triggered by voltage-dependent membrane oscillations that have been shown to be dependent on sodium conductances^15^. We demonstrate that only thalamic stimulation was able to increase the oscillation frequencies, with no significant effect mediated by cortical stimulation. Thus, our data suggest that thalamic stimulation move the cells to a more excitable state allowing the neurons to firing continuously. The relative higher abundance of AMPA-mediated current found at thalamic synapses provides evidence supporting this idea. Indeed, thalamic stimulation might increase the number of membrane oscillations, as well as the firing rate of FSI, by modulating sodium conductances through large AMPA-mediated currents^40^. Contrarily, cortical stimulation did not affect membrane oscillation frequencies nor action potential threshold. In attempt to find a mechanistic correlate to the different subthreshold oscillation frequency and spiking patterns induced by cortical vs. thalamic stimulation, we found that mGlu1 receptors are critically involved in the thalamic response of FSIs, but not in the responses to cortical activation. These findings are consistent with previous evidence showing that, although FSIs express both group I mGlu receptors, only mGlu1 mediates membrane and synaptic responses^25^.

### Functional implication of distinct firing modulation by cortex and thalamus

Cortical layers project onto the striatum in a highly topographically organised manner providing motor and cognitive information^35^. Conversely, the intralaminar thalamic nuclei drive sensory, attentional and salience information^4^,^6^. In the last few years growing interest has been paid to study how functional interplay between cortico- and thalamostriatal pathways may modulate striatal circuit and in particular MSNs excitability^6^,^8^,^41^. Interestingly, recent investigations proved that thalamic and cortical information may be conveyed to MSNs through both a direct excitatory input or by an indirect pathway mediated by striatal cholinergic interneurons^6^,^7^.

We provide evidence for a novel role for this class of interneurons by which thalamo- and corticostriatal inputs can affect striatal output. FSIs make inhibitory synapses onto hundreds of surrounding MSNs by means of a dense but restricted axonal field and they provide powerful and temporally coded inhibitory influence over MSN spike initiation^12^,^42^,^43^. Moreover FSIs appear to preferentially contact striatonigral rather than striatopallidal MSNs^44^ and their firing desynchronization is sufficient to alter balanced firing between D1 and D2 carrying MSNs in a striatal network model^45^. Collectively, such evidence suggests that modulation of firing activity of FSI could strongly impact onto striatal circuit.

Of interest, although our data on short-term synaptic plasticity clearly provide evidence for differences between corticostriatal and thalamostriatal synapses onto FSIs, however, they also highlight an interesting similarity between FSIs and MSNs^6^. Indeed, corticostriatal inputs to both MSNs and FSIs are facilitatory, whereas thalamostriatal inputs onto both neuron subtypes result in a synaptic depression. This finding may imply that, regardless the neuronal subtype involved, the two glutamatergic inputs convey distinct activation signals that require a concerted activation of the cellular type targeted. Besides basic synaptic properties, one of the mechanisms that contributes to dissecting the properties of these two pathways is the involvement of mGlu1 receptors. Thalamic and cortical synapses have a different extrasynaptic expression and localization of mGlu receptors^4^,^25^ and our data clearly show that mGlu1 receptors play a crucial role in selectively modulating thalamostriatal but not corticostriatal input onto FSIs.

Cortical stimulation activates GABAergic interneurons before both ChIs and MSNs^46^, thus corticostriatal synapses, by increasing the intermittent activity of FSI, could contribute to refine the action selection process during behavioural tasks^47^. However, the exact sequence of cellular targets activated by thalamostriatal inputs is still unknown, although we speculate that thalamostriatal synapses exhibiting high release probability and more prominent AMPA-mediated currents are able to quickly modulate firing of FSI triggering a fast, massive and temporally coded inhibition of neighboring MSNs. This powerful mechanism may represent another way by which thalamic inputs convey salient sensory stimuli into striatum^48^,^49^.

Growing evidence strongly suggests that structural or functional deficits of FSIs are implicated in human diseases. A reduction in FSIs number has been reported in Tourette’s syndrome^50^, Huntington’s disease^51^,^52^ and paroxysmal dystonia^53^. Moreover, pharmacologically-induced hypofunction of striatal FSIs produces dystonia-like movements in rodents^54^. Similarly, in dopamine-depleted mice, FSIs imbalances MSNs spiking activity^55^. Thus, a better understanding on how cortical and thalamostriatal inputs refine striatal output through FSIs represents an important step towards dissecting basal ganglia activity in both physiological and pathological conditions.

## Methods

### Animals

The Animal Care and Use Committee of University of Rome “Tor Vergata” approved all experiments. Animal experiments were carried out in accord with EC, Internal Institutional Review Committee, EU directive and Italian rules (86/609/EEC; D.Lvo 116/1992, 26/2014, 14/2014, EU 63/2010 respectively). All members of the staff involved in the experiments have undergone a specific training for handling and working with rodents. All efforts were made to reduce the number of animals used and minimize their suffering. All experiments were performed on transgenic mice (B6.Cg-Tg(Thy1-COP4/EYFP)18Gfng/J; control strain: https://www.jax.org/strain/007612; The Jackson Laboratory; n = 25) expressing the light-activated ion channel, Channelrhodopsin-2 (ChR2) fused to Yellow Fluorescent Protein under the control of the mouse thymus cell antigen 1 (*Thy1*) promoter. Selective expression of ChR2 has been demonstrated both in cortical neurons of layer V and thalamic neurons (Fig. 1a,b)^56^,^57^. C57BL/6J mice were also used for control experiments (n = 14).

### Slice preparation

Mice were killed by cervical dislocation under ether anesthesia and the brain immediately removed from the skull. A parahorizontal brain slice preparation (250 μm) was used in order to better preserve the connectivity of thalamic and cortical fibers^6^,^8^,^35^ (Fig. 1a,c). Slices were cut with a vibratome in Krebs’ solution containing (in mM): 126 NaCl, 2.5 KCl, 1.3 MgCl_2_, 1.2 NaH_2_PO_4_, 2 CaCl_2_, 10 glucose, and 18 NaHCO_3_), bubbled with 95% O_2_ and 5% CO_2_ as described previously^8^. After 30–60 min recovery at room temperature (RT), individual slices were transferred to a recording chamber (0.5–1 ml volume) and continuously superfused with oxygenated Krebs’ medium (2.5–3 ml/min, 32–35 °C). Recordings were performed on individual FSI visualized by means of IR-DIC videomicroscopy with an Olympus BX-51WIF camera/controller system (Olympus). For voltage-clamp experiments pipettes (3–5 MΩ) were filled with Cs^+^internal solution containing (in mM): 120 CsMeSO_3_, 15 CsCl, 8 NaCl, 10 TEA-Cl, 10 HEPES, 0.2 EGTA, 2 Mg-ATP, and 0.3 Na-GTP, pH 7.3 adjusted with CsOH. For current clamp experiments, pipettes (3–5 MΩ) were filled with K^+^internal solution containing (in mM) 135 KMeSo_4_, 5 KCl, 0.5 CaCl_2_, 5 HEPES, 5 EGTA, 2 Mg-ATP, and 0.3 Na-GTP, pH 7.3 adjusted with KOH. Experiments were done at 32–35 °C. During glutamatergic fiber stimulation, picrotoxin (10 μM) was added to the bathing solution to block GABA_a_ mediated currents. Recordings were made using a Multiclamp 700b amplifier (Molecular Devices, Sunnyvale, CA). Signals were digitized at 10–20 KHz using Digidata 1322 acquisition board. Data digitized were collected using pCLAMP 9.2 software (Molecular Devices, Sunnyvale, CA) and stored for off-line analysis. Membrane currents were continuously monitored and access resistance measured in voltage-clamp was in the range of 5–30 MΩ before electronic compensation (60–80% routinely used). Series resistance for whole cell recordings (20–50 MΏ) were compensated at the amplifier. Changes in series resistance higher of 25% caused discard of data collected.

### Stimulation and electrophysiological recording protocols

Glutamatergic fibers were stimulated either electrically or optically. Electrical stimulation (20–200 μs) was performed by placing a steel concentric electrode (FHC Inc) either in layer V of the cortex for activation of the cortical projection or close to the border of thalamic reticular nucleus for thalamic fiber stimulation (Fig. 1c). Optical stimulation (3 ms) of corticostriatal and thalamostriatal afferents was performed using pE-2 LED systems (CoolLED, UK), consisting of a 470 nm LED mounted on Olympus BX-51WIF microscope. Light power at microscope objective exit was 2–20 mW/mm^2^. Spot light was made by by utilizing a diaphragm placed along the light path. Slices were illuminated from the bottom and a round spot was created with a radius of approximately 200 μm in order to avoid light spread to unwanted brain structure, and delivered on cortical layer V (corticostriatal synapses stimulation) and on thalamus (thalamostriatal synapses stimulation) (Fig. 1a–c). Electrical and optical stimulation intensity was calculated in the following mode. A stimulation able to induce an action potential was considered as maximal. Each stimulation pulse was calculated as 5, 10, 25, 50 75 and 90% of maximal stimulation. The coefficient of variation (CV) for a given measured variable was defined as the ratio between the standard deviation and the average value. For short-term synaptic plasticity experiments two stimulating pulses were consecutively given at 25, 50, 100, 200, 500, 1000 and 5000 ms interval (up to 20 pulses, 10 s interval). Train pulses consisted of 5 pulses at 20 Hz repeated every 30 s for up to 5 times. Release probability was calculated by a nonstationary fluctuation analysis of evoked EPSCs to compute the variance of >30 consecutive EPSCs after cortical or thalamic stimulation. EPSCs were obtained with the extracellular Ca^2+^/Mg^2+^concentrations at normal levels and then at 1 mM Ca^2+^and 4 mM Ca^2+^. EPSC variance and mean amplitude were calculated as described^17^. NMDA/AMPA ratios were calculated as following: average waveforms from the –70 and +40 mV (holding potential) steps were baseline subtracted and measured. The time of the peak current at –70 mV, considered to be fully mediated by AMPAR, was used to establish the time window for measuring the AMPA peak at +40 mV. The decay to baseline of the AMPA current at –70 was used to select a time window for measurement of the NMDA current; a 10-ms measurement window beginning 40 ms after the stimulus was used. This current was designated as the NMDA measurement. (*I*_NMDA at +40 mV_/*I*_AMPA at +40 mV_) was taken as the NMDA/AMPA ratio. To calculate the decay times of averaged currents, curves were fitted with a double exponential equation of the form *I*(*t*) = *If* exp(−*t*/τ*f*) + *Is* exp(−*t*/τ*s*), where *If* and *Is* are the amplitudes of the fast and slow decay components, and τ*f* and τ*s* are their respective decay time constants. To compare distinct decay times we used a weighted mean decay time constant τ*w* = [*If*/(*If* + *Is*)]/*tf* + [*Is*/(*If* + *Is)*]/*ts*^58^. AMPA-mediated quantal events were recorded 50 ms after each stimulus (5 pulse, 25 Hz, delivered at 0.01 Hz for both cortical and thalamic stimulation) for a maximum period of a 300 ms^8^. External Ca^2+^ was replaced with Sr^2+^ (2 mM) and MK-801 (30 μM) was added to block NMDA currents. All events were analyzed (Minianalysis; Synaptosoft) as described previously^59^. Sub-threshold membrane oscillation was defined as the most negative membrane potential level at which spontaneous variation of the membrane potentials (>1 mV, <5 ms) took place in both depolarizing and hyperpolarizing directions^15^. The interval between two consecutive peaks was considered as a cycle. To measure the oscillation frequency, episodes in which at least four consecutive cycles were present, and peaks were clearly identifiable, were considered. Mann-Whitney test was used to find statistical significance.

### Confocal imaging of brain slices

Following electrophysiological recordings coronal or parahorizontal brain slices were fixed in 4% paraformaldehyde in 0.12 M phosphate buffer at pH 7.4 overnight. Slices were then washed in PBS and mounted with Vectashield® mounting medium on plus polarized glass slides (Super Frost Plus Thermo Scientific) and coverslipped. All images were acquired with a LSM700 Zeiss confocal laser scanning microscope (Zeiss, Göttingen, Germany). Fluorescent signals were recorded using 488 nm as wavelength for excitation. To obtain images of slices derived from Th1ChR2-EYFPmice we used low magnification objectives, 1.25× and 5×, with an additional digital zoom factor of 0.5. The confocal pinhole was kept at 1, the gain and the offset were adjusted to prevent saturation of the signal. Single section confocal images (1024 × 1024) were collected and exported in TIFF file format. Background subtraction was performed on all images by means of ImageJ software.

### Data analysis and statistics

Data analysis was performed with Clampfit 9.2 (Molecular Devices), Origin 8.0 (Microcal), Prism 5.3 (GraphPad), MiniAnalysis (Synaptosoft). Box plots and bar graphs were used for graphic illustration of result. In box plots superimposed circles represent single experiments. Non-matched samples were analyzed with the nonparametric Mann–Whitney rank sum test. Matched samples were analyzed with Wilcoxon signed rank test. Data are represented as means ± SEM. A p value < 0.05 vas set as significance level for all statistic tests.

## Additional Information

**How to cite this article**: Sciamanna, G. *et al.* Optogenetic stimulation reveals distinct modulatory properties of thalamostriatal vs corticostriatal glutamatergic inputs to fast-spiking interneurons. *Sci. Rep.* **5**, 16742; doi: 10.1038/srep16742 (2015).
